# A Remotely Delivered GLP-1RA–Supported Specialist Weight Management Program in Adults Living With Obesity: Retrospective Service Evaluation

**DOI:** 10.2196/72577

**Published:** 2025-06-30

**Authors:** Rebecca Richards, Michael Whitman, Gina Wren, Peta Campion

**Affiliations:** 1 Second Nature London United Kingdom; 2 Nuffield Department of Primary Care Health Sciences University of Oxford Oxford United Kingdom

**Keywords:** obesity, weight management, glucagon-like peptide-1 receptor agonist, GLP-1RA, semaglutide, digital health, remote health care delivery, telemedicine, behavioral intervention, weight loss, cost-effectiveness, service evaluation, real-world evidence

## Abstract

**Background:**

Limited access to specialist weight management services restricts the implementation of novel pharmacotherapies for obesity such as glucagon-like peptide-1 receptor agonists (GLP-1RAs) in the UK National Health Service (NHS). Second Nature, a commercial digital health company, offers a remotely delivered program combining a GLP-1RA medication (semaglutide) with digital behavioral support, potentially providing a scalable solution. However, evidence for long-term effectiveness in this real-world context is limited.

**Objective:**

This study aimed to evaluate the 12-month effectiveness, feasibility, acceptability, and potential cost-effectiveness of the remotely delivered, semaglutide-supported weight management program by Second Nature.

**Methods:**

This retrospective service evaluation analyzed data from participants who initiated the program between September and December 2023. The primary outcome was weight change at 12 months among participants with available data (completers). Secondary outcomes included retention, program engagement (measured by views of the Home screen in the app), behavioral changes, side effects, participant experience (qualitative analysis), and a comparative cost analysis against an NHS specialist weight management service. An “active subscription” was defined as maintaining a paid subscription for the full 12-month period. Descriptive statistics and paired 2-tailed *t* tests evaluated outcomes.

**Results:**

Data from 341 participants were included at baseline (282/341, 82.7% women; mean age 49, SD 11.1 years; mean baseline BMI 37.9, SD 6.9 kg/m^2^). At 12 months, 39.6% (135/206) maintained an active subscription, while 60.4% (206/341) became inactive. Weight data at 12 months were available for 179 participants (52.5% of the baseline cohort; 100% of active and 19.4% of inactive participants). Among completers who maintained an active subscription, the mean weight loss was 20.0 kg (SD 8.7 kg; *P*<.001), representing 19.1% of starting weight. Overall, 77.7% (139/179) of completers achieved ≥10% weight loss and 61.5% (110/179) achieved ≥15%. Program engagement declined over time. Side effects also decreased, with 69.6% (81/116) of respondents reporting none by month 12. Most participants completing the 12-month survey reported positive (41/120, 34.2%) or neutral (68/120, 56.7%) experiences.

**Conclusions:**

This evaluation suggests that remotely delivered GLP-1RA–supported weight management can achieve significant weight loss in participants remaining engaged for 12 months. However, the high rate of withdrawal limits generalizability. The program appears feasible, acceptable, and potentially cost-effective for completers. Further research, ideally in public health care settings using intent-to-treat analyses, is needed to confirm clinical outcomes, assess sustained results, and understand factors influencing retention.

## Introduction

### Background

Obesity remains a global health crisis, affecting 890 million adults worldwide and contributing to diseases such as type 2 diabetes, cardiovascular disease, and certain cancers [1]. In the United Kingdom, projections suggest that by 2050, the annual cost of obesity to the National Health Service (NHS) could reach £9.7 billion (US $12.1 billion), with wider societal expenses approaching £49.9 billion (US $62.4 billion) [2]. Although lifestyle interventions form the foundation of obesity treatment, their long-term effectiveness varies considerably, with more than half of lost weight typically regained within 2 years of weight loss [3].

Clinical trials of glucagon-like peptide-1 receptor agonists (GLP-1RAs), including semaglutide and tirzepatide, have demonstrated significant weight reductions, ranging from 14.9% to 20.9% over 68 to 72 weeks, respectively [4-6]. However, significant weight regain occurs following treatment discontinuation, with STEP-1 trial participants regaining approximately two-thirds of lost weight within 1 year of withdrawing from semaglutide (2.4 mg) and lifestyle intervention [7]. The National Institute for Health and Care Excellence (NICE) guidance recommending tirzepatide for obesity management alongside lifestyle modification [8] has no maximum treatment duration, suggesting potential long-term use. This necessitates the development of sustainable, cost-effective approaches to implement prolonged GLP-1RA treatment and lifestyle modifications.

To date, eligible people living with obesity in the United Kingdom have primarily accessed GLP-1RAs and lifestyle support through NHS specialist weight management services (SWMSs). SWMSs typically offer multidisciplinary team (MDT) support including dietitians, physicians, psychologists, and physiotherapists, often through a tiered system requiring general practitioner (GP) referrals [9]. However, because of underfunding, these services face significant challenges including long waiting lists, understaffing, and limited treatment flexibility, hindering timely access and potentially impacting outcomes [8,9]. In response to these issues and the anticipated demand for newer medications such as tirzepatide, NICE guidance suggests prescribing, monitoring, and multidisciplinary behavioral support could potentially be implemented in primary care [8].

However, concerns exist regarding the capacity of primary care services, particularly GPs, to absorb this additional workload. Despite manufacturer surveys indicating GP interest [8], recent data highlight increasing patient caseloads (17% more patients per GP since 2015) and appointment demands (5.46 million more appointments in February 2024 vs 2019), alongside a 3% decrease in full-time equivalent GPs [10,11]. Significant service transformation appears necessary to meet this demand within the NHS.

The NICE Health Technology Evaluation 14 (HTE14) guidance specifically highlights the potential role of remote interventions in increasing access to GLP-1RA medication and associated support [9]. Emerging evidence suggests that remotely delivered SWMSs could offer a scalable approach to address service provision gaps [9,10,12]. For example, a preliminary 12-week service evaluation of a remotely delivered program combining GLP-1RA (semaglutide) with app-based behavioral support (the same program evaluated in this study) showed that participants lost an average of 6.4% of body weight [12]. This earlier evaluation also suggested feasibility, with relatively low withdrawal rates over 12 weeks, manageable side effects, and generally positive or neutral participant experiences [12]. Furthermore, early economic modeling within the NICE HTE14 process indicated potential cost-effectiveness for remote SWMSs compared with traditional in-person services [9]. However, longer-term (≥12 months) real-world evidence for remotely delivered GLP-1RA–supported weight management interventions is crucial to substantiate their potential role in expanding access to NICE-recommended obesity treatments.

### Objective

This retrospective service evaluation of a remotely delivered, semaglutide-supported weight management program by Second Nature was conducted to evaluate its effectiveness, feasibility, acceptability, and potential cost-effectiveness over a 12-month period in a real-world commercial setting.

## Methods

### Overview

This retrospective analysis used routinely collected service data from participants of a remotely delivered, semaglutide-supported commercial weight management program provided by Second Nature Healthy Habits Ltd, a UK-based digital health company. The study focused on participants who initiated the program between September and December 2023. As a service evaluation of existing data, the analysis primarily reflects outcomes among those who remained in the program and provided data at 12 months (ie, a completer analysis), particularly for the primary effectiveness outcome.

The core intervention, delivery methods, and data collection procedures were consistent with our previously reported 12-week evaluation [12]. Program adaptations were implemented following NICE guidance for tirzepatide, which, unlike semaglutide at the time, had no recommended treatment duration limit [8]. Consequently, the intervention was modified from a fixed duration to an open-ended format supporting individualized treatment periods. Program phases were redesignated from “transition” and “maintain” to “progress” and “momentum” to reflect observed participant behavior patterns, as significant weight loss often continued during these later phases. While this cohort used semaglutide exclusively and was followed for 12 months, these structural modifications enabled extended support for ongoing GLP-1RA–assisted weight management where clinically appropriate. No participants in this cohort received tirzepatide.

### Ethical Considerations

This evaluation study met Health Research Authority and Medical Research Council criteria for service evaluation [13]: it assessed current care using existing, anonymized data from an established intervention, did not involve any change to standard practice, and did not involve randomization or a control group. The Health Research Authority explicitly states that “because both audit and service evaluation are considered part of usual professional practice, they are exempt from oversight processes that govern research—that is, they do not require review by a research ethics committee” [14]. The study analyzed anonymized, non-NHS data that could not identify individuals, and thus did not require formal ethics approval (Multimedia Appendix 1). Participants were not compensated for taking part in the study. Participants consented to the research use of anonymized data through acceptance of the program’s privacy policy at registration. The evaluation adhered to General Data Protection Regulations, maintaining participants’ right to request data deletion.

### Participants

Retrospective data were extracted from the Second Nature database in December 2024 for all participants (N=341) who initiated the intervention between September and December 2023. This timeframe allowed for a full 12-month potential follow-up period. All participants meeting the eligibility criteria during this period were included in the baseline analysis.

Eligible participants were adults aged 18 to 75 years living with obesity (BMI ≥30 kg/m^2^) who had access to and could use a smartphone or tablet. Exclusion criteria included current or a history of eating disorders; pregnancy, breastfeeding, or actively trying to conceive; specific allergies to medication ingredients; concurrent use of certain medications (eg, other GLP-1RAs and insulin); and specific health conditions (eg, current or historical thyroid cancer, active cancer, inflammatory bowel disease, pancreatitis, severe liver or kidney disease, heart failure, multiple endocrine neoplasia type 2, gallbladder issues, or diabetic retinopathy). People living with type 2 diabetes were eligible if they were not taking any of the excluded medications (eg, metformin was allowed).

Participants were allowed to transition to a version of the digital program that focused only on behavioral support without pharmacotherapy; they were transferred to a separate intervention and were not included in this analysis.

Participants self-referred to the program, joining through digital marketing channels such as Google or Facebook, through word-of-mouth referrals from friends or family, or through guides written by Second Nature that were discovered through search engine results. The cost to the participants varied between £179 (US $224) and £299 (US $374) per month, depending on the dose of semaglutide prescribed.

### Intervention

#### Overview

The intervention combined GLP-1RA medication (semaglutide) prescribed by independent prescribing pharmacists from Pharmalogic Chemist (a UK-based pharmacy provider) with Second Nature’s digital weight management platform. Following clinical assessment and approval, participants received semaglutide, injection safety equipment, wireless weighing scales (Renpho Elis 1), and physical program materials (a FAQ leaflet, nutrition guidelines, and a recipe book) delivered to their address.

Support was provided by registered dietitians or nutritionists (“health coaches”) with the Health and Care Professions Council or Association for Nutrition, respectively. Nutritionist-led coaching was supervised by a dietitian. Health coaches completed training covering medication management (including side-effect support), program delivery protocols, clinical governance, and safeguarding. The training was developed by senior MDT members and approved by the lead prescriber, with ongoing quality assurance via coaching reviews. The MDT included registered dietitians, nutritionists, clinical pharmacists, and a safeguarding lead.

#### App-Based Program

The digital program, delivered via Second Nature’s smartphone and web applications, consisted of 5 phases: prepare (0-12 d), adapt (17 wk), grow (17 wk), progress (17 wk), and momentum (≥17 wk). This 17-week phase was designed to provide sufficient time for adapting and building new habits. The program used evidence-based behavior change techniques throughout all phases to help participants adapt to medication, adopt healthier long-term lifestyle habits (nutrition, physical activity, sleep, and stress management), and support sustainable weight management.

Educational content (nutrition, medication or side-effect management, behavioral psychology, physical activity, sleep hygiene, mental well-being, and stress management) was delivered through short daily articles. The app integrated self-monitoring tools for weight (via connected scales), activity, and sleep. The recommended frequency for self-monitoring weight was individualized for each participant based on their health coach’s assessment of their progress, adherence pattern, and personal preferences. However, general guidelines were given to record weight at a consistent time each week, typically in the morning after waking and before eating or drinking. Physical activity was tracked through daily steps using the participant’s smartphone or fitness tracker (no additional device was provided by the program), and participants were encouraged to self-monitor changes in sleep quality, duration, and energy levels. The program was developed by an MDT following NICE guidance for obesity management and behavior change principles [9], building upon Second Nature’s established digital weight management program [12,15,16].

Participants received feedback on their progress through multiple channels. The app displayed weight trends visually through graphs showing progress toward individualized weight loss goals. Weekly automated messages summarized their weight change to date and the percentage of their starting weight lost. Health coaches provided personalized feedback on progress during one-to-one messaging conversations, discussing weight trajectory, dietary choices, physical activity achievements, and addressing any challenges or plateaus.

Goal setting was an integral component of the program. At initiation, participants established an individualized target weight loss goal in consultation with their health coach, typically aiming for 15% to 20% of starting weight over 12 months based on GLP-1RA clinical trial outcomes. Weekly step goals were automatically suggested based on the participant’s recent activity levels, gradually increasing as their activity improved. Sleep goals were more personalized, with participants encouraged to establish consistent sleep routines, aiming for 7 to 9 hours per night.

Participants received personalized support via asynchronous one-to-one messaging with their health coach during business hours, alongside moderated peer support groups within the app. Health coaches posted structured content weekly and responded to messages within specified timeframes. Interactions with the health coaches were ad hoc and based on participant needs, questions, and the educational content provided (covering topics such as the GLP-1RA medication [semaglutide], side effects, nutrition, physical activity, sleep, stress management, and mental well-being). If a participant became at risk of disengaging (defined as 4 d since their last Home screen view), health coaches were prompted to message them proactively. Program engagement was monitored through automated tracking of app interactions (see the Effectiveness Measures section). A notification system prompted the completion of mandatory monthly check-ins and weight measurements.

#### Medication

Semaglutide was prescribed with a 4-weekly dose titration schedule until the optimal therapeutic effect was achieved with minimal side effects. Medication management followed standardized safety protocols, with pharmacist oversight of initiation and repeat prescribing based on monthly digital monitoring surveys and consultations when clinically indicated. By month 12, all participants with active subscriptions (100%) had reached the maximum 2.4 mg dose.

Participants were guided on how to take the medication through educational resources, such as manufacturer’s guides and injection demonstration videos [17]. The Second Nature web and smartphone application served as a central hub for ongoing support, featuring a dedicated medication “toolbox” and relevant daily content.

Safety monitoring comprised mandatory monthly digital surveys reviewed by pharmacists for prescription decisions. The platform enabled real-time adverse event reporting through health coach messaging, with 24-hour response times (business days). Medical concerns were escalated to prescribing teams via secure messaging, with pharmacist responses provided within 4 hours (business days). The system monitored safety indicators including BMI and rapid weight changes. Weight was tracked via Bluetooth-connected scales, and participants were encouraged to record their weight weekly for safety and to measure progress toward goals.

A tiered adverse event management protocol was implemented. Mild symptoms were initially managed through health coach–led lifestyle modifications, with a pharmacist reviewing if symptoms persisted beyond 7 days. Moderate or severe symptoms prompted immediate pharmacist assessment to determine intervention pathways. Emergency protocols directed participants to urgent care services for acute medical events. All clinical interactions were documented systematically across coaching and clinical platforms.

### Data Collection and Measures

#### Overview

Data were analyzed from participants who initiated the intervention between September and December 2023. Participants were categorized at 12 months based on their subscription status. Active participants maintained a paid program subscription for the full 12 months. Inactive participants cancelled their self-funded subscription before the 12-month mark. Cancellation terminated medication provision and health coach support but maintained limited app access (educational resources and weight tracking).

Baseline characteristics (height, weight, age, and gender) were collected via web-based onboarding surveys and verified by customer support staff. Identity verification used LexisNexis third-party authentication, requiring a minimum of 2 database matches across Electoral Roll, Companies House, Experian, and Equifax records. Authentication failure criteria included age <18 years, deceased status, or birth data inconsistencies. Data collection comprised Bluetooth-transmitted weight measurements, monthly surveys capturing information on clinical suitability behavioral changes and participant experience, and digital platform engagement metrics. All data were stored in Second Nature’s secure database.

#### Effectiveness Measures

The primary outcome was mean weight change (kg and % of starting weight) only at 12 months, calculated using the last recorded weight between months 11 and 13 (ie, day 330-420) compared with baseline weight. This analysis was performed on the subset of participants with available 12-month weight data (completers). The weight reading recorded for inactive participants was their last available weight reading. Secondary effectiveness outcomes included the proportion of completers achieving clinically significant weight loss thresholds: ≥10% and ≥15% of baseline weight. Weight measurements were recorded using the provided wireless scales. Participants were instructed on proper use (firm, flat surface, morning, postvoiding, and consistent day). A validation algorithm flagged potentially erroneous readings based on expected ranges, prompting user confirmation or contact with support staff.

#### Cost Measures

The cost-effectiveness of the intervention was evaluated through a comparative cost analysis between the commercial medication-supported intervention and NHS SWMSs. Cost data for NHS SWMSs were derived from NICE HTE14 guidance, economic modeling, and resource impact assessments [9,18], combining expert consultation with 2022 Personal Social Services Research Unit costs. Analysis of 12 SWMS experts (11 providing detailed data) showed that standard care includes multidisciplinary support with weekly to fortnightly dietetic appointments for 2 to 3 months, followed by monthly follow-ups. Consultant reviews occur every 3 to 6 months, while psychological support comprises 6-week group sessions or bi- or triweekly individual therapy. Physiotherapy is provided at 1- to 3-month intervals. Attendance rates range from 60% to 90% (median 70%-75%), with higher rates (approximately 90%) reported for consultant and psychology appointments [9].

Additional costs were incorporated from NICE TA6179 guidance on tirzepatide for managing overweight and obesity [8], which provided broader cost ranges for implementing wraparound support alongside medication in community or primary care settings.

Using the NICE Impact Assessment Tool (IAT) [18], we analyzed costs per 100,000 population. The eligible proportion for semaglutide obesity treatment and SWMSs was set to the default value suggested in the calculator (9.50064%). On the basis of SWMS expert feedback from the NICE HTE14 supporting documentation [9], we estimated that 30% of the eligible population would prefer a remote option, with 75% ultimately starting the service based on data from Second Nature’s NHS-delivered SWMS. We used base-case costs from NICE HTE14 guidance in the “Standard care (without app)” tab of the IAT and confidential costs for Second Nature’s intervention [9,18].

For commercial sensitivity, percentage reductions were rounded to the nearest 10% using a relative cost-reporting approach. Costs were standardized to per-patient figures for 12 months of treatment. Percentage differences were calculated between the intervention and both primary and secondary care standard models detailed in NICE HTE14 guidance and the tirzepatide technology appraisal [8,9,18]. The analysis focused on direct intervention costs only, excluding long-term health improvement savings.

#### Behavioral Changes

Lifestyle-related behavioral changes, including diet, fruit and vegetable intake, and physical activity levels, were assessed through a series of mandatory web-based surveys administered at months 1, 3, 6, and 12 of the program. Participants were asked to report their daily vegetable and fruit consumption by responding to the question, “How many different vegetables or fruits do you eat a day on average?” Responses were recorded numerically to reflect the average number of different items consumed daily. To evaluate physical activity levels, participants were asked, “How many times do you exercise each week on average?” This question required participants to report the average number of exercise sessions completed per week. In addition, participants were asked about their meal preparation habits with the question, “How many times do you cook each week on average?” Responses were recorded as the number of cooking sessions per week. Finally, to assess dietary modifications, participants responded to the question, “What changes have you made to your diet over the last few weeks?” The number of dietary changes was reported numerically and analyzed at each survey time point.

#### Acceptability

Secondary outcomes included the acceptability of the intervention, which was assessed using the following measures.

#### Retention and Cancellation Reasons

Withdrawal was defined as participants cancelling their subscription to the program. The number of people who maintained an active subscription or cancelled their subscription was recorded, and reasons for cancelations were collected through an in-app cancellation form.

#### Program Engagement

Engagement with the app program was assessed using Home screen views, which measured the number of times the app was opened. The duration of program participation was calculated as the number of months between the participant’s intervention start date and the effective cancelation date (defined as the final day in a month when the participant had full access to all intervention features).

#### Feasibility

##### Side Effects

Participants were emailed a mandatory web-based check-in survey at months 1, 3, 6, and 12 to collect information on medication side effects. Participants could select from a drop-down menu of 15 possible side effects, “none,” or “other.” Selecting “other” prompted a mandatory free-text response for the description. If a side effect was selected, participants were asked an additional question: “Are these side effects tolerable?” with response options of “yes” or “no.” If “no” was selected, participants were asked, “Do you want advice or support with these side effects from our clinical team?” with the same response options.

##### Medication Administration

Responses to a question from the mandatory web-based check-in survey administered at months 1, 3, 6, and 12 were extracted to assess the acceptability of medication administration. The question, “Are you having any difficulty injecting yourself?” included 3 preset responses: “No”; “Yes, and it means I’m not injecting once a week on the same day”; and “Yes, but I’m still injecting once a week on the same day.”

##### Participant Experience

Participants’ experiences with the program were assessed by analyzing free-text responses to the question, “How have you found the last few weeks of the program?”

### Statistical Analysis

Analyses were performed using R statistical software (version 4.3.3). For effectiveness, we calculated mean weight loss and SD. We evaluated statistical significance using paired 2-tailed *t* tests (*P*<.05) and also calculated the proportions of participants achieving ≥10% and ≥15% weight loss thresholds. Statistical analysis included descriptive statistics for baseline characteristics and primary weight change outcomes. We also descriptively analyzed weight change by subscription status (“active” vs “inactive” paid subscriptions). Program retention was analyzed by calculating active subscription rates and categorizing withdrawal reasons. Monthly behavioral changes, side effects, and participant experiences were analyzed as proportions of total survey respondents. Program engagement was assessed through mean monthly Home screen views. Cost comparisons used the NICE IAT to model costs per 100,000 population, assuming 9.50064% eligibility, 30% digital service uptake, and 75% initiation rate. We standardized annual per-patient costs and calculated percentage differences between the intervention and NHS standard care models [18].

### Qualitative Analysis

Content analysis was conducted to analyze the free-text responses to the monthly survey question, “How have you found the last few weeks of the program?” For each question, at each time point, the responses were coded and organized into higher-level categories by MW. These frameworks were then refined based on the protocol outlined in a previous study to create 9 higher-level categories that captured key themes in participants’ experiences [12]. Each response could be categorized into multiple themes to reflect the complexity of participants’ experiences. For example, when a participant submitted a free-text response of “ok,” “fine,” “not bad,” “nothing to report,” or “same as before” to the “How have you found the last few weeks on the program?” question, it would be categorized as a “neutral experience.”

## Results

### Baseline Characteristics and Retention

**Figure 1:**
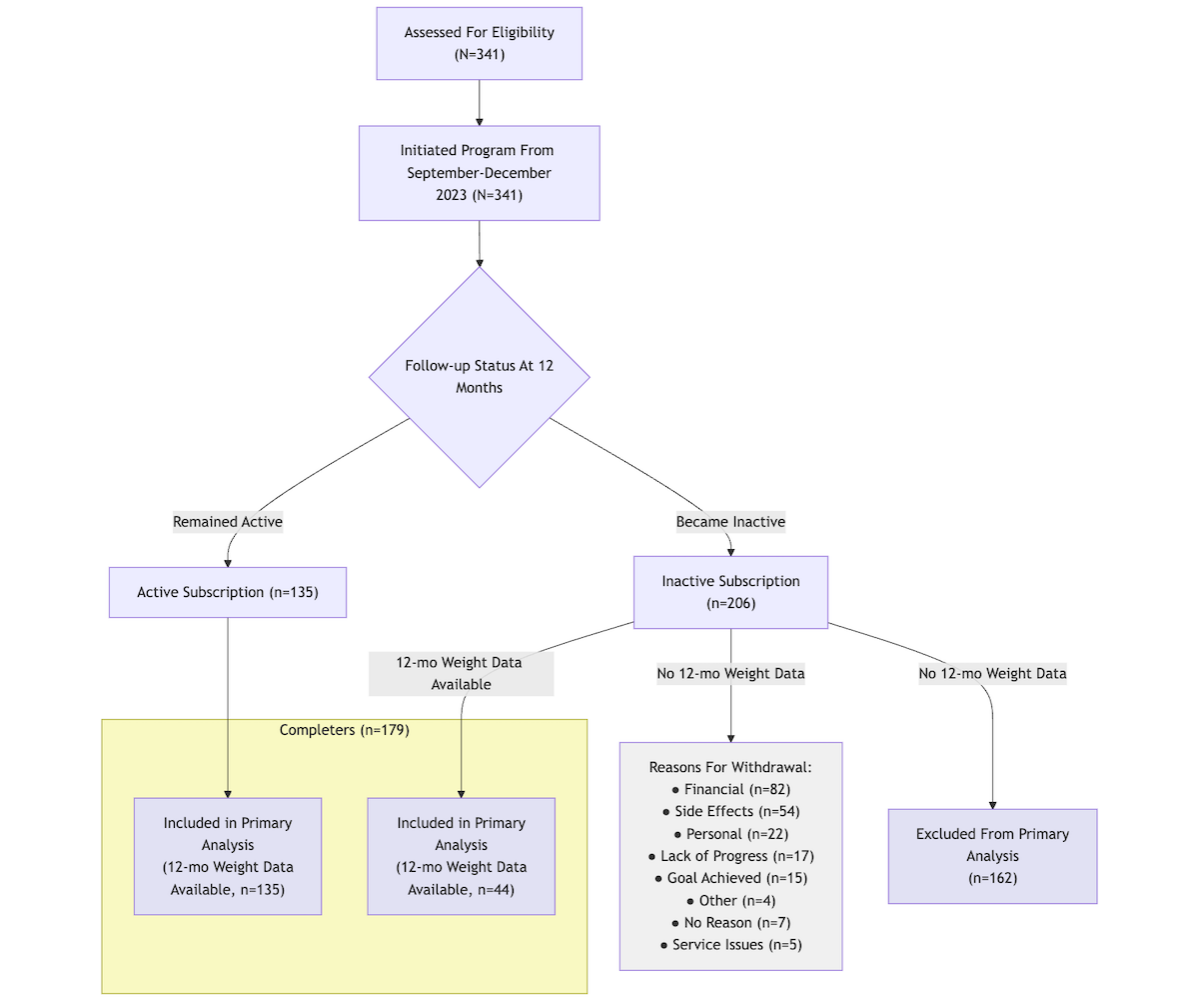
Participant flow through the 12-month semaglutide-supported weight management program, showing retention, data availability, and withdrawal reasons.

A total of 341 participants initiated the program between September and December 2023 and were included in the baseline analysis (Figure 1). Baseline characteristics are presented in Table 1. The majority were women (282/341, 82.7%), with a mean age of 48.2 (SD 11.7) years and a mean baseline BMI of 37.7 (SD 6.9) kg/m^2^. There were no statistically significant differences in baseline characteristics between those who remained active and those who became inactive at 12 months (data not shown; *P*>.05 for all comparisons).

**Table 1:** Baseline characteristics of all participants: those with active subscriptions at 12 months and those with inactive subscriptions at 12 months.

Characteristics	All participants (N=341)	Active participants at 12 mo (n=135)	Inactive participants at 12 mo (n=206)
Age (y), mean (SD)	48.2 (11.7)	49 (11.1)	47.7 (12.1)
Female, n (%)	282 (82.7)	111 (82.2)	171 (83)
Baseline weight (kg), mean (SD)	105.7 (21.3)	106.6 (20.5)	105.1 (21.8)
Baseline BMI (kg/m²), mean (SD)	37.7 (6.9)	37.9 (6.9)	37.6 (6.9)

At the 12-month time point, of the 341 participants, 135 (39.6%) maintained an active paid subscription. The remaining 206 (60.4%) cancelled their subscriptions and became inactive. The mean duration of program participation for the entire cohort was 8.2 (SD 4.2) months. For active participants, the mean duration was 12.5 (SD 0.6) months (reflecting ongoing participation at data extraction), while for inactive participants, the mean duration before cancellation was 5.4 (SD 3.0) months. Weight data at 12 months were available for 179 (N=341, 52.5%) participants of the baseline cohort. This included all 135 (100%) active participants and 44 (n=206, 21.4%) inactive participants who continued to record weight data after cancellation.

### Effectiveness

#### Weight Loss

Among the 179 participants with 12-month weight data (completers), the mean weight loss from baseline was 18.1 kg (SD 9.5 kg; *P*<.001), corresponding to 17.3% (SD 8.9%) of starting weight. Weight loss differed by subscription status among completers. Participants with an active subscription at 12 months (135/179, 75%) achieved a mean weight loss of 20.0 kg (SD 8.7 kg; *P*<.001), representing 19.1% (SD 8.0%) of their starting weight. Participants who were inactive but provided 12-month weight data (44/179, 24%) achieved a mean weight loss of 12.4 kg (SD 10.6 kg; *P*<.001), representing 11.9% (SD 9.8%) of their starting weight. Overall, among the 179 completers, 139 (77.7%) achieved ≥10% weight loss and 110 (61.5%) achieved ≥15% weight loss at 12 months. Among 135 active completers, 116 (85.9%) achieved ≥10% weight loss and 94 (69.6%) achieved ≥15% weight loss. Among 44 inactive completers, 23 (52.3%) achieved ≥10% weight loss and 16 (36.4%) achieved ≥15% weight loss.

#### Cost Compared With Usual Care

The external assessment group for the NICE HTE14 guidance estimated the cost of delivering a secondary care SWMS to be £1796 (US $2245) per patient per year [18]. Depending on the specific implementation requirements for an NHS health care setting, we estimated that the cost of Second Nature’s service would be between 30% and 40% of the current standard care for NHS SWMSs. In addition, costs provided in the supporting documentation for the NICE guidance on tirzepatide for managing overweight and obesity estimated the cost of an MDT-supported weight management intervention delivered in the community or primary care settings at £868.21 to £1239.21 (US $1085.13 to $1549.01) per patient per year [8]. The intervention costs were estimated to be 30% to 90% of the costs for these hypothetical models of delivering an in-person or hybrid MDT in a community or primary care setting.

### Behavioral Changes

When asked about fruit and vegetable consumption, most participants (151/280, 54%) at month 1 and (62/119, 52.1%) at month 12 reported consuming 3 to 4 different items per day across all time points (Tables 2 and 3). The proportion consuming ≥5 items remained relatively stable from 63 (n=280, 22.4%) at month 1 to 28 (n=119, 23.5%) at month 12.

**Table 2:** Responses to a lifestyle-related behavioral survey by month (% of total respondents; months 1-6).

Category and subcategory		Month										
		1 (n=367)		2 (n=308)		3 (n=272)		4 (n=250)		5 (n=220)		6 (n=191)
**Fruit and vegetable consumption (items per d), n (%)**												
	0	4 (1.1)	4 (1.3)		3 (1.1)		3 (1.2)		1 (0.5)		2 (1)	
	1-2	81 (22.4)	71 (23)		71 (25.7)		60 (24.3)		53 (24.1)		45 (23.4)	
	3-4	195 (54)	166 (53.7)		141 (50.7)		132 (53.4)		115 (52.3)		102 (53.1)	
	≥5	81 (22.4)	68 (22)		62 (22.5)		52 (21.1)		52 (23.2)		43 (22.4)	
**Exercise (times/wk), n (%)**												
	0	78 (21.6)	48 (15.5)		28 (9.7)		20 (7.9)		14 (6.5)		13 (6.6)	
	1-2	152 (42.1)	139 (44.8)		120 (43.2)		115 (45.3)		101 (46.5)		78 (39.8)	
	3-4	91 (25.2)	91 (29.4)		96 (34.5)		86 (33.9)		77 (35)		73 (37.2)	
	≥5	40 (11.1)	32 (10.3)		35 (12.6)		33 (13)		26 (12)		32 (16.3)	
**Cooking frequency, n (%)**												
	Never	5 (1.4)	3 (1)		4 (1.5)		2 (0.8)		2 (0.9)		1 (0.5)	
	Less than once per wk	7 (1.9)	3 (1)		9 (3.3)		6 (2.4)		5 (2.3)		10 (5.2)	
	Once per wk	10 (2.7)	9 (2.9)		8 (3)		12 (4.8)		6 (2.7)		11 (5.8)	
	Few times per wk	90 (24.5)	91 (29.5)		69 (25.5)		75 (30)		71 (32.4)		57 (29.8)	
	Nearly every day	133 (36.2)	117 (38)		109 (40.2)		96 (38.4)		85 (38.4)		80 (41.9)	
	Every day	122 (33.2)	85 (27.6)		73 (26.6)		59 (23.6)		51 (23.3)		32 (16.8)	

**Table 3:** Responses to a lifestyle-related behavioral survey by month (% of total respondents; months 7-12).

Category and subcategory		Month						
		7 (n=181)	8 (n=127)	9 (n=138)	10 (n=133)	11 (n=136)		12 (n=119)
**Fruit and vegetable consumption (items/d), n (%)**								
	0	2 (1.1)	2 (1.5)	1 (0.7)	1 (0.8)	2 (1.4)		1 (0.8)
	1-2	37 (20.2)	30 (22.9)	24 (17.5)	27 (20.3)	24 (17.4)		28 (23.5)
	3-4	95 (51.9)	63 (48.1)	73 (53.3)	74 (55.6)	83 (60.1)		62 (52.1)
	≥5	49 (26.8)	36 (27.5)	39 (28.5)	31 (23.3)	29 (21)		29 (23.5)
**Exercise (times/wk), n (%)**								
	0	9 (4.9)	10 (7.6)	7 (5.1)	9 (6.7)		11 (8)	9 (7.6)
	1-2	73 (39.7)	50 (38.2)	49 (35.8)	55 (41)		58 (42)	47 (39.5)
	3-4	75 (40.8)	51 (38.9)	57 (41.6)	47 (35.1)		50 (36.2)	48 (39.5)
	≥5	27 (14.7)	20 (15.3)	24 (17.5)	23 (17.2)		19 (13.8)	16 (13.4)
**Cooking frequency, n (%)**								
	Never	1 (0.6)	2 (1.6)	0 (0)	1 (0.8)		1 (0.7)	1 (0.8)
	Less than once per wk	5 (2.8)	2 (1.6)	4 (2.9)	4 (3)		6 (4.4)	3 (2.5)
	Once per wk	6 (3.3)	5 (3.9)	8 (5.8)	8 (6)		8 (5.9)	7 (5.9)
	Few times per wk	60 (33.1)	46 (36.2)	41 (29.7)	39 (29.3)		37 (27.2)	34 (28.8)
	Nearly every day	73 (40.3)	38 (29.9)	58 (42)	56 (42.1)		59 (43.4)	52 (43.2)
	Every day	36 (19.9)	34 (26.8)	27 (19.6)	25 (18.8)		25 (18.4)	22 (18.6)

The proportion exercising 3 to 4 times per week or every day increased, whereas the proportion exercising 1 to 2 times per week stayed relatively consistent. In addition, the number of participants who reported no exercise per week decreased from 60 (n=280, 21.5%) participants in month 1 to 9 (n=119, 7.6%) in month 12. An increase in cooking frequency was observed over 12 months, except for those who reported cooking “every day” at baseline, which decreased from 92 (n=280, 33%) participants to 22 (n=119, 18.6%) participants, and those who “never” cooked, which decreased from 4 (n=280, 1.4%) participants to 1 (n=119, 0.8%) participant.

### Acceptability

#### Retention and Cancellation Reasons

Of the 341 participants included in the analysis, 135 (39.6%) maintained an active paid subscription after 12 months. The remaining 206 (60.4%) cancelled their subscriptions before 12 months. However, 44 (21.4%) of these participants continued to record weight data. Cancellation reasons for those who withdrew were categorized as follows: financial concerns (70/206, 34%), medical issues and side effects (49/206, 23.8%), no reason given (29/206, 14.1%), personal circumstances (19/206, 9.2%), perceived lack of weight loss progress (16/206, 7.8%), perceived achievement of desired weight goal (15/206, 7.3%), support and service issues (5/206, 2.4%), and other (eg, where ambiguous information was provided in response to determine an accurate reason for cancellation, such as “I can not be on the injections any longer”; 3/206, 1.5%). The mean program participation was 8.2 (SD 4.2) months overall, with active participants averaging 12.5 (SD 0.6) months at the point of data collection and inactive participants averaging 5.4 (SD 3.0) months. All participants who maintained an active subscription submitted weight readings. Of the 206 participants who withdrew before the 12-month mark, 44 (21.4%) participants submitted their weight readings.

#### Program Engagement

**Figure 2:**
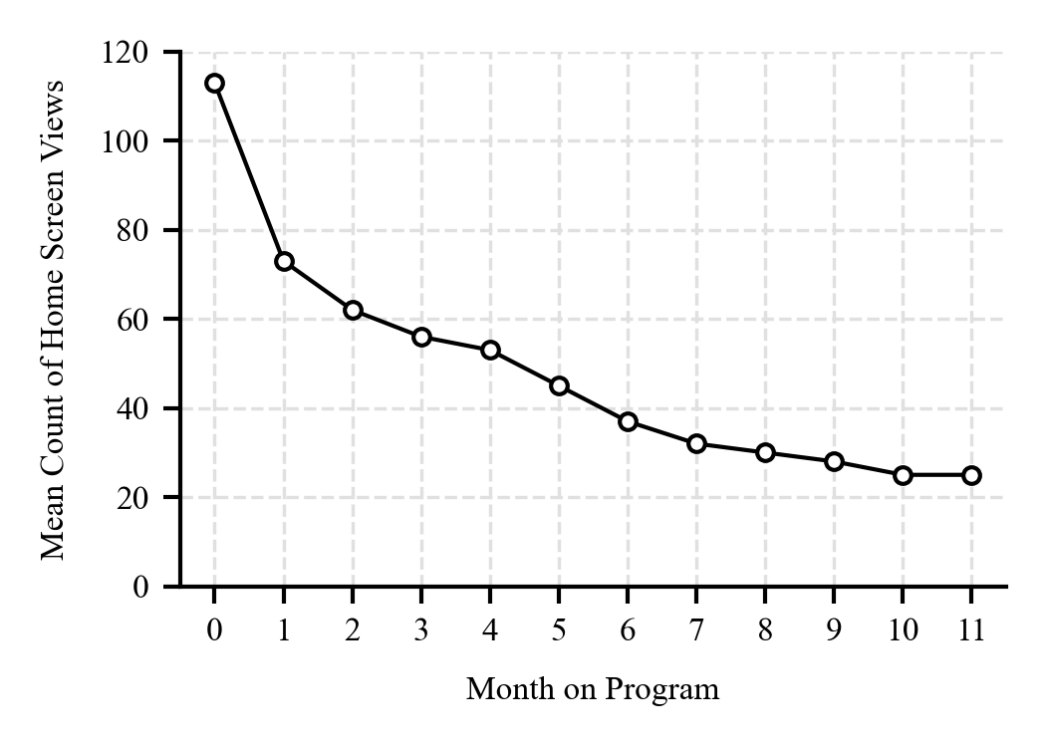
The mean number of application Home screen views by month on the program among the active participants.

The mean Home screen view count started at 113 (SD 78.4) in the first month, reduced to 73 (SD 65) in the second month, and then steadily declined each month to 25 (SD 29.06) by month 12 (Figure 2).

### Feasibility

#### Side Effects

In the first month, the most reported side effects were feeling sick (90/313, 28.8%), constipation (80/313, 25.6%), and feeling more tired than usual (57/313, 18.2%); 154 (n=313, 49.2%) participants reported no side effects at this stage. After 12 months, 80 (n=116, 69%) participants reported no side effects, and the most common side effects were constipation (18/116, 15.5%), feeling sick (8/116, 7%), and hair loss (7/116, 6%; Table 4).

**Table 4:** Percentage of participants reporting side effects by survey month (% of total respondents).

Side effect	Month, n (%)											
	1 (n=313)	2 (n=297)	3 (n=264)	4 (n=239)	5 (n=216)	6 (n=186)	7 (n=179)	8 (n=130)	9 (n=136)	10 (n=132)	11 (n=135)	12 (n=116)
Constipation	80 (25.6)	91 (30.6)	83 (31.4)	65 (27.2)	53 (24.5)	35 (18.8)	35 (19.6)	21 (16.2)	25 (18.4)	27 (20.5)	21 (15.6)	18 (15.5)
Diarrhea	27 (8.6)	31 (10.4)	29 (11)	34 (14.2)	28 (13)	17 (9.1)	17 (9.5)	8 (6.2)	12 (8.8)	12 (9.1)	8 (5.9)	7 (6)
Dry skin and mouth	1 (0.3)	0 (0)	0 (0)	0 (0)	0 (0)	0 (0)	0 (0)	0 (0)	0 (0)	0 (0)	0 (0)	0 (0)
Fast, deep breathing	0 (0)	0 (0)	1 (0.4)	0 (0)	0 (0)	0 (0)	0 (0)	0 (0)	0 (0)	0 (0)	0 (0)	0 (0)
Feeling anxious	3 (1)	10 (3.4)	4 (1.5)	7 (2.9)	1 (0.5)	4 (2.2)	2 (1.1)	2 (1.5)	1 (0.7)	2 (1.5)	1 (0.7)	2 (1.7)
Feeling dizzy	13 (4.2)	12 (4)	12 (4.5)	9 (3.8)	8 (3.7)	4 (2.2)	4 (2.2)	2 (1.5)	5 (3.7)	3 (2.3)	2 (1.5)	6 (5.2)
Feeling more tired than usual	57 (18.2)	64 (21.5)	64 (24.2)	56 (23.4)	41 (19)	30 (16.1)	18 (10.1)	16 (12.3)	10 (7.4)	9 (6.8)	8 (5.9)	6 (5.2)
Feeling sick	90 (28.8)	99 (33.3)	94 (35.6)	67 (28)	64 (29.6)	38 (20.4)	24 (13.4)	17 (13.1)	13 (9.6)	17 (12.9)	13 (9.6)	8 (6.9)
Fever or high temperature	0 (0)	1 (0.3)	0 (0)	0 (0)	0 (0)	0 (0)	0 (0)	0 (0)	0 (0)	0 (0)	0 (0)	0 (0)
Flushed face	0 (0)	0 (0)	1 (0.4)	0 (0)	0 (0)	0 (0)	0 (0)	0 (0)	0 (0)	0 (0)	0 (0)	0 (0)
Hair loss	7 (2.2)	3 (1)	5 (1.9)	3 (1.3)	3 (1.4)	3 (1.6)	9 (5)	10 (7.7)	8 (5.9)	10 (7.6)	10 (7.4)	7 (6)
Headaches	32 (10.2)	25 (8.4)	25 (9.5)	17 (7.1)	20 (9.3)	8 (4.3)	5 (2.8)	6 (4.6)	2 (1.5)	5 (3.8)	2 (1.5)	4 (3.4)
Heartburn or indigestion	42 (13.4)	59 (19.9)	56 (21.2)	42 (17.6)	32 (14.8)	18 (9.7)	14 (7.8)	7 (5.4)	10 (7.4)	8 (6.1)	8 (5.9)	5 (4.3)
Muscle stiffness or aches	1 (0.3)	0 (0)	0 (0)	0 (0)	0 (0)	0 (0)	1 (0.6)	0 (0)	0 (0)	0 (0)	0 (0)	0 (0)
No side effects	154 (49.2)	100 (33.7)	80 (30.3)	91 (38.1)	88 (40.7)	99 (53.2)	98 (54.7)	76 (58.5)	89 (65.4)	82 (62.1)	87 (64.4)	80 (69)
None of the above	4 (1.3)	2 (0.7)	3 (1.1)	3 (1.3)	2 (0.9)	3 (1.6)	2 (1.1)	1 (0.8)	1 (0.7)	1 (0.8)	1 (0.7)	0 (0)
Reactions in the injection site	3 (1)	2 (0.7)	4 (1.5)	3 (1.3)	4 (1.9)	0 (0)	2 (1.1)	1 (0.8)	4 (2.9)	5 (3.8)	7 (5.2)	1 (0.9)
Severe stomach pain	2 (0.6)	5 (1.7)	2 (0.8)	5 (2.1)	5 (2.3)	1 (0.5)	0 (0)	1 (0.8)	1 (0.7)	1 (0.8)	3 (2.2)	1 (0.9)
Tender or swollen stomach	6 (1.9)	7 (2.4)	11 (4.2)	4 (1.7)	2 (0.9)	2 (1.1)	1 (0.6)	1 (0.8)	1 (0.7)	1 (0.8)	1 (0.7)	0 (0)
Vomiting	8 (2.6)	14 (4.7)	20 (7.6)	20 (8.4)	11 (5.1)	9 (4.8)	11 (6.1)	3 (2.3)	6 (4.4)	6 (4.5)	3 (2.2)	2 (1.7)
Yellowing of the skin or eyes	0 (0)	0 (0)	0 (0)	0 (0)	1 (0.5)	0 (0)	0 (0)	0 (0)	0 (0)	0 (0)	0 (0)	0 (0)

#### Medication Administration

Most participants reported no difficulties with medication administration. At month 1, of the 315 participants, 310 (98.4%) reported no difficulties, and only 5 (1.5%) indicated they needed support with injecting. At months 2 and 3, a total of 297 (n=297, 100%) participants reported no difficulties. Small fluctuations were observed at month 4 by 1 (n=242, 0.4%) participant and month 5 by 1 (n=216, 0.5%) participant where participants reported injection difficulties. At month 7, only 1 (n=182, 0.5%) participant reported difficulties. From month 8 to month 12, all participants consistently reported no difficulties with injection administration. Throughout the program, all participants who reported difficulties still maintained their prescribed weekly injection schedule.

### Participant Experience

The participant responses were categorized into 9 distinct areas: change in appetite, illness, intervention benefits, negative experience, neutral experience, other priorities, positive experience, and slow progress. Initially, most participants reported positive experiences (211/320, 65.9% at month 1), but this steadily declined to just over a third (41/120, 34.2%) by month 12. Conversely, neutral experiences increased from 15.9% (51/320) at month 1 to 56.7% (68/120) at month 12 (Table 5). Reported intervention benefits were reduced throughout the program, decreasing from 17.2% (55/320) at month 1 to 3.3% (4/120) at month 12. Similarly, reports of appetite changes declined from 8.4% (27/320) to <1%. Negative experiences and reduced effectiveness were consistently reported by 1% (1/120) of participants.

**Table 5:** Percentage of participants reporting different experiences throughout by program month (% of total respondents).

Category	Month, n (%)											
	1 (n=320)	2 (n=301)	3 (n=274)	4 (n=242)	5 (n=218)	6 (n=192)	7 (n=182)	8 (n=130)	9 (n=137)	10 (n=133)	11 (n=137)	12 (n=120)
Change in appetite	27 (8.4)	32 (10.6)	25 (9.1)	13 (5.4)	9 (4.1)	6 (3.1)	6 (3.3)	2 (1.5)	3 (2.2)	4 (3)	1 (0.7)	1 (0.8)
Illness	6 (1.9)	4 (1.3)	4 (1.5)	4 (1.7)	4 (1.8)	3 (1.6)	6 (3.3)	1 (0.8)	2 (1.5)	0 (0)	0 (0)	0 (0)
Intervention benefits	55 (17.2)	32 (10.6)	41 (15)	27 (11.2)	22 (10.1)	20 (10.4)	15 (8.2)	10 (7.7)	9 (6.6)	12 (9)	7 (5.1)	4 (3.3)
Negative experience	2 (0.6)	1 (0.3)	1 (0.4)	2 (0.8)	0 (0)	2 (1)	0 (0)	0 (0)	0 (0)	0 (0)	0 (0)	0 (0)
Neutral experience	51 (15.9)	46 (15.3)	58 (21.2)	84 (34.7)	94 (43.1)	90 (46.9)	84 (46.2)	73 (56.2)	66 (48.2)	76 (57.1)	82 (59.9)	68 (56.7)
Other	13 (4.1)	6 (2)	5 (1.8)	3 (1.2)	3 (1.4)	3 (1.6)	4 (2.2)	3 (2.3)	4 (2.9)	2 (1.5)	0 (0)	2 (1.7)
Other priorities	5 (1.6)	8 (2.7)	5 (1.8)	7 (2.9)	8 (3.7)	1 (0.5)	5 (2.7)	2 (1.5)	1 (0.7)	2 (1.5)	1 (0.7)	4 (3.3)
Positive experience	211 (65.9)	183 (60.8)	143 (52.2)	111 (45.9)	86 (39.4)	70 (36.5)	65 (35.7)	39 (30)	53 (38.7)	37 (27.8)	46 (33.6)	41 (34.2)
Reduced effectiveness or slow progress	1 (0.3)	0 (0)	0 (0)	0 (0)	2 (0.9)	0 (0)	0 (0)	1 (0.8)	0 (0)	0 (0)	1 (0.7)	0 (0)

## Discussion

### Principal Findings

This 12-month retrospective service evaluation provides real-world insights into a remotely delivered, semaglutide-supported commercial weight management program. The primary finding is that participants who completed 12 months of the program (179/341, 52.5%) of the initial cohort achieved substantial and clinically significant weight loss, with a mean reduction of 17.3% (18.1 kg). Those who maintained an active subscription throughout achieved even greater mean weight loss (19.1%, 20 kg). However, these findings must be interpreted cautiously because of the high withdrawal rate, with 47.5% (162/341) of participants providing no 12-month weight data, and 60.4% (206/341), cancelling their subscriptions, meaning the results primarily reflect outcomes in a self-selected group of completers.

The program demonstrated feasibility regarding medication administration and side-effect management among those who remained. Side effects were most common early in the program but decreased significantly over time, with the vast majority of participants reporting no side effects after 12 months. Self-injection was not a significant barrier. Acceptability, as judged by participant experience surveys among respondents, remained largely positive or neutral at 12 months; although positive sentiment decreased over time, neutral responses increased. Engagement with the digital platform, measured by Home screen views, showed a typical pattern of decline over the 12 months. Encouragingly, positive lifestyle changes related to exercise and cooking frequency appeared to be sustained or improved over the 12 months among survey respondents.

The intervention’s estimated cost was substantially lower than traditional NHS SWMS models, suggesting the potential for cost savings if implemented within a public health care system. However, this requires confirmation through formal health economic analysis in that context.

### Comparison With Prior Work

The mean weight reduction of 19.1% (SD 8.0%) for participants with an active subscription observed in this study are comparable with outcomes reported in both controlled trials and real-world evaluations of GLP-1RA medications, respectively [19-21]. For example, reviews of the semaglutide STEP randomized controlled trials showed mean weight reductions of 14% to 17% over 68 weeks [19], while tirzepatide trials demonstrated weight loss of 14.7% to 20.9% across populations with and without diabetes [20]. However, direct comparisons are limited by differences in study design (randomized controlled trials vs real-world evaluation), participant populations (trial eligibility vs commercial users), follow-up duration, the intensity of lifestyle support, and analytic approach (intent-to-treat [ITT] vs completer analysis).

Comparison with other real-world, remotely delivered, GLP-1RA–supported behavioral change programs with completer analyses are more relevant. Two real-world studies in the United States reported 12-month weight reductions with semaglutide when combined with remote support: one found a reduction of 13.8% to 15.3% and another reported a reduction of 13.9% [21,22]. This study demonstrated higher rates of substantial weight loss, with 77.7% (139/179) of participants achieving ≥10% and 61.5% (110/179) achieving ≥15% at 12 months, compared with 38.7% and 18.4%, respectively, in comparable remotely delivered program in the United States [22].

An important finding relates to the inactive participants who provided 12-month weight data (44/206, 21% of those who cancelled). These individuals, who stopped the paid program (including medication and coaching) after an average of 5.4 months, still maintained a mean weight loss of 11.9% at the 12-month mark. This observation requires cautious interpretation because of the small sample size and potential for selection bias. However, it aligns with recent evidence suggesting less weight regain after stopping semaglutide when combined with behavioral support compared with medication withdrawal alone [7,23]. The STEP 1 trial extension showed a regain of about two-thirds (approximately 11.6% absolute weight) of the weight 1 year after stopping semaglutide and intensive behavioral therapy [7], whereas another study reported only 3% regain 12 months after semaglutide cessation in participants who continued a nutritional program [23]. Our finding, while preliminary and based on limited data, suggests the potential role of the initial behavioral intervention component in mitigating weight regain after program cessation.

Program retention rates in remotely delivered weight management services vary substantially. The 12-month retention rate of 39.6% (135/341) observed in this study falls between previously reported rates ranging from 22.7% at 5 months [24] to 58% at 9 months [25] for other GLP-1RA–supported remotely delivered interventions, although structural and financial differences limit direct comparisons. The observed decline in app engagement (Home screen views) is typical for digital health interventions [22,26] and does not necessarily equate to a lack of benefit but highlights the need for strategies to maintain long-term engagement, if required. The improvement in side-effect tolerability over time is consistent with clinical trial data [19] and is reassuring for long-term use. However, the fact that 26% (49/206) of participants who withdrew cited side effects suggests that early intolerance remains a significant barrier for a subset of users, contributing to the lower prevalence of side effects observed in completers (a form of survivorship bias).

It is possible that the behavioral support provided in this study increased and supported the maintenance of exercise, dietary quality, and home cooking to support and enhance the benefits of the GLP-1RA medication. For example, exercise and maintaining dietary quality have been recommended during the use of GLP-1RAs to retain muscle mass during weight loss [27-31]. However, comparison with existing literature is constrained because of the lack of comprehensive behavioral data in GLP-1RA research. Typically, studies of GLP-1RA medication have focused on caloric intake and satiety measures rather than broader lifestyle modifications [32]. Therefore, further research on the impact of behavioral support on GLP-1RA outcomes is needed.

### Cost Comparison and Implications

On the basis of NICE modeling data [9,18], this remotely delivered intervention appears to be substantially less costly than traditional secondary care SWMSs. Calculations using the NICE IAT suggest potential direct cost reductions of 40% to 50% per 100,000 population (£1.5-1.8 million; US $1.9-2.25 million) compared with the HTE14 standard care estimate [18]. While these are preliminary estimates based on modeling assumptions and confidential commercial costs, they support the hypothesis that remote delivery could offer a more resource-efficient way to provide the necessary wraparound support for GLP-1RA therapy, potentially freeing up NHS resources or enabling wider access. A formal health economic evaluation within an NHS setting is required to confirm these potential savings.

### Strengths and Limitations

The strengths of this study include its relatively large initial sample size, 12-month follow-up duration, and real-world setting, providing insights beyond those from controlled trials. The use of connected scales offers objective weight data, and the collection of data on retention, engagement, side effects, behaviors, and experience provides a multifaceted evaluation.

However, the findings of this study have several limitations. The observational design without a comparator group inherently limits the ability to definitively attribute observed weight loss solely to the intervention, as secular trends or unmeasured participant factors could play a role. This design issue is compounded by the substantial participant withdrawal rate (206/341, 60.4%) by 12 months. Consequently, the primary effectiveness analysis relies on data from completers (179/341, 52.5% of the initial cohort), a group likely to differ systematically from those who dropped out (eg, in motivation, medication tolerability, or initial success). This reliance on a completer analysis significantly limits the generalizability of the effectiveness findings. An ITT analysis with appropriate imputation for missing data, while beyond the scope of this service evaluation, would be necessary to estimate effectiveness across all individuals who initiated the program.

Generalizability is also influenced by the nature of the participant cohort. As it is a commercial program requiring self-payment, participants primarily enrolled via digital marketing, search engines, or word-of-mouth referrals. This self-selection process likely yielded a group with sufficient financial resources and motivation, potentially differing from the broader NHS patient population. The high proportion citing cost as a reason for withdrawal underscores this potential selection bias and suggests that retention patterns and possibly outcomes might differ substantially in a publicly funded model. Relatedly, while initial health conditions were reviewed during onboarding (partly via NHS Summary Care Record access), detailed baseline data on comorbidities (including type 2 diabetes status), ethnicity, or concurrent medications were not accessible for this specific analysis, restricting a full understanding of the cohort’s characteristics and factors influencing outcomes.

Methodological limitations also extend to data collection and measurement. Behavioral changes were assessed using self-reported data from nonvalidated questionnaires, introducing potential recall and social desirability bias. Furthermore, the absence of baseline behavioral data precludes a direct assessment of change from preintervention habits. Program engagement was measured by Home screen views, which is an unvalidated metric of engagement. More granular data on the use of specific features such as coaching interactions or self-monitoring tool entries were not included in this analysis but may offer avenues for future investigation. Although connected scales provided objective weight data, the real-world setting meant weighing frequency and conditions were not strictly standardized.

The evaluation also lacked data on broader clinical outcomes (eg, metabolic parameters) or patient-reported outcomes, such as quality of life. In addition, participants could transition to a separate nonmedication program, and these individuals were excluded; thus, the analysis does not capture outcomes for those who may have stopped medication but continued behavioral support under a different structure. Moreover, while the last available weight was used for inactive participants with 12-month data, the specific weight trajectory immediately preceding withdrawal for the majority who became inactive earlier was not analyzed.

Finally, although the 12-month duration exceeds that of many digital health evaluations, it may be insufficient to assess the long-term sustainability of weight loss and behavioral changes, particularly concerning potential medication cessation effects. Future research should aim to address these limitations, for instance, through designs incorporating matched controls or randomization, standardized measurements using validated instruments, comprehensive baseline and outcome data collection, ITT analyses, and extended follow-up periods.

### Conclusions

This real-world service evaluation suggests that a remotely delivered, GLP-1RA–supported weight management program is an effective, feasible, acceptable, and cost-effective approach for achieving weight loss and improving health behaviors in people living with obesity. These programs could address the current service provision issues with specialist in-person services, but further research is needed to evaluate their use within a public health care setting, assess their impact on other clinical and psychological measures, and evaluate sustained effects with continuation and discontinuation of the medication.

## Appendix

Multimedia Appendix 1 UK Health Research Authority ethics exemption: result—not research.

## Abbreviations

GLP-1RA: glucagon-like peptide-1 receptor agonist
GP: general practitioner
HTE14: Health Technology Evaluation 14
IAT: Impact Assessment Tool
ITT: intent-to-treat
MDT: multidisciplinary team
NICE: National Institute for Health and Care Excellence
SWMS: specialist weight management service
