# Supplementary material for: A Remotely Delivered GLP-1RA–Supported Specialist Weight Management Program in Adults Living With Obesity: Retrospective Service Evaluation
Source: JMIR Form Res. 2025 Jun 30;9:e72577. doi: 10.2196/72577 (PMC12260464; doi:10.2196/72577)
Supplement: Multimedia Appendix 1 [file formative_v9i1e72577_app1.pdf]

Go straight to content.

Medical Research Council

NHS Health Research Authority

Do I need NHS REC review?

To print your result with title and IRAS Project ID please enter your details below:

Title of your research:

A Remotely Delivered, GLP-1RA-Supported Specialist Weight Management Program: 12 Month Outcomes From a Retrospective Service Evaluation

IRAS Project ID (if available):

You have answered **'No'** to the question "Is your study research" which indicates that **you do not need NHS REC review**.

This tool only considers whether NHS REC review is required, it does not consider whether other approvals are needed. You should check whether other approvals are required for your study.

Note: **Post Market Surveillance** is NOT usually considered research. However, there are some circumstances where NHS REC review may be required. Please follow the link below to start again and select YES at the first question to determine if your post market surveillance requires NHS REC review.

To understand how research is defined, please visit the **Is my study research?** decision tool.

Follow this link to start again.

Print This Page

NOTE: If using Internet Explorer please use browser print function.

About this tool **Feedback** **Contact** **Glossary** **Algorithm** **Accessibility**
